# Effectiveness and Feasibility of Taxing Salt and Foods High in Sodium: A Systematic Review of the Evidence

**DOI:** 10.1093/advances/nmaa067

**Published:** 2020-06-20

**Authors:** Rebecca Dodd, Joseph Alvin Santos, Monique Tan, Norm R C Campbell, Cliona Ni Mhurchu, Laura Cobb, Michael F Jacobson, Feng J He, Kathy Trieu, Sutayut Osornprasop, Jacqui Webster

**Affiliations:** The George Institute for Global Health, University of New South Wales, Sydney, Australia; The George Institute for Global Health, University of New South Wales, Sydney, Australia; Wolfson Institute of Preventive Medicine, Barts and The London School of Medicine and Dentistry, Queen Mary University of London, London, UK; University of Calgary, Calgary, Alberta, Canada; The George Institute for Global Health, University of New South Wales, Sydney, Australia; Resolve to Save Lives, An Initiative of Vital Strategies, New York City, NY, USA; Center for Science in the Public Interest, Washington, DC, USA; Wolfson Institute of Preventive Medicine, Barts and The London School of Medicine and Dentistry, Queen Mary University of London, London, UK; National Institute for Health Innovation, University of Auckland, Auckland, New Zealand; The George Institute for Global Health, University of New South Wales, Sydney, Australia; Global Practice on Health, Nutrition, and Population, The World Bank, Washington, DC, USA; The George Institute for Global Health, University of New South Wales, Sydney, Australia

**Keywords:** salt tax, salt reduction, salt intake, hypertension, salt, sodium, best buys

## Abstract

Diets high in salt are a leading risk for death and disability globally. Taxing unhealthy food is an effective means of influencing what people eat and improving population health. Although there is a growing body of evidence on taxing products high in sugar, and unhealthy foods more broadly, there is limited knowledge or experience of using fiscal measures to reduce salt consumption. We searched peer-reviewed databases [MEDLINE, Embase, Cochrane Central Register of Controlled Trials (CENTRAL), and the Cochrane Database of Systematic Reviews] and gray literature for studies published between January 2000 and October 2019. Studies were included if they provided information on the impact on salt consumption of: taxes on salt; taxes on foods high in salt, and taxes on unhealthy foods defined to include foods high in salt. Studies were excluded if their definition of unhealthy foods did not specify high salt or sodium. We found 18 relevant studies, including 15 studies reporting the effects of salt taxes through modeling (8), real-world evaluation (4), experimental design (2), or review of cost-effectiveness (1); 6 studies providing information relevant to country implementation of salt taxes; and 2 studies reporting stakeholder perceptions toward salt taxation. Although there is some evidence on the potential effectiveness and cost-effectiveness of salt taxation, especially from modeling studies, uptake of salt taxation is limited in practice. Some modeling studies suggested that food taxes can have unintended outcomes such as reduced consumption of healthy foods, or increased consumption of unhealthy, untaxed substitutes. In contrast, modeling studies that combined taxes for unhealthy foods with subsidies found that the benefits were increased. Modeling suggests that taxing all foods based on their salt content is likely to have more impact than taxing specific products high in salt given that salt is pervasive in the food chain. However, the limited experience we found suggests that policy-makers favor taxing specific products.

## Introduction

Globally, unhealthy diet is the leading risk for premature death and the second leading risk for disability (1). Excessive salt intake is the most harmful of the dietary risk factors, associated with >3 million deaths and the loss of 70 million disability-adjusted life years (DALYs) in 2017 (1, 2). It is a well-established cause of high blood pressure and increases the risk of cardiovascular disease and kidney disease (3). Although some scientists continue to produce and cite studies with paradoxical findings that conflict with the evidence base (4–6), multiple independent review processes have concluded that most national population salt intakes are too high and that this creates serious health problems (7–10). The current WHO recommendation is that salt (sodium chloride) intake should be <5 g/d for adults (11). Salt is comprised of sodium plus chloride, and it is sodium that is harmful to health (12). Although foods contain other forms of sodium, such as sodium bicarbonate, salt accounts for 90% of the sodium people consume. Therefore, in this article we refer to sodium in food as “salt.”

Many people are poorly informed on recommended levels of salt intake and struggle to understand nutrition labeling (13, 14). Accordingly, efforts to reduce salt intake in the population are more likely to be effective when they encompass a range of interventions and include population-wide measures such as reformulation and pricing policies, alongside policies that support informed consumer choice such as front-of-pack warnings, interpretative labeling (e.g., star ratings), and limits on advertising (15–17).

Among the population-wide measures, fiscal measures and price controls can reduce demand for unhealthy products by making them more expensive, and thus less appealing to consumers. Systematic reviews on sugar tax, fat tax, and sweetened beverage taxes found good evidence that these led to healthier purchases (18–23). The WHO goal, endorsed by all WHO member states, is to reduce salt consumption by 30% by 2025 (24), through comprehensive salt reduction strategies. Fiscal measures are recognized as one important approach, with WHO recommending “appropriate fiscal policies and regulation” to reduce salt intake (24), a call echoed by health care professionals and academics (25).

This study considers both the effectiveness and the feasibility of salt taxation as a policy measure to improve population health. We provide a narrative summary of the evidence on salt taxation, based on a systematic review of available studies, and provide an overview of “real-world” implementation of salt taxes to date. Whereas other systematic reviews have considered sugar, fat, and broader “unhealthy food” taxes, to our knowledge, no study has reviewed the evidence on impact and implementation of salt taxation.

## Methods

### Search strategy

The review protocol for the systematic review was registered at the International Prospective Register of Systematic Reviews (PROSPERO; registration number CRD42019150732).

A search for peer-reviewed literature published between January 2000 and October 2019 was conducted in MEDLINE, Embase, Cochrane Central Register of Controlled Trials (CENTRAL), and Cochrane Database of Systematic Reviews. Our 20-y search period ensured a comprehensive overview of recent literature and was seen as appropriate given increasing global attention to noncommunicable diseases (NCDs), including those caused by poor diet, over the last 2 decades, culminating in a series of UN high-level meetings on NCDs, beginning in 2011 (26).

Keywords comprised terms related to salt or sodium, and taxation or fiscal measures. **Supplemental Table 1** lists the full search strategy in MEDLINE, which was adapted for the other databases. A gray literature search applying the same search terms was conducted in OpenGrey, Google Scholar, WHO regional websites, Caribbean Food and Nutrition Institute, World Action on Salt and Health, Centers for Disease Control and Prevention, Public Health Agency of Canada, and Institute of Medicine resources. Specifically, we searched the gray literature for information on real-world implementation on taxation, including information on the scope and structure of salt tax regimes and any evidence evaluating implementation. No language or study type restrictions were applied during the search.

We used a 2015 review titled “Salt reduction initiatives around the world” (15) to identify an initial list of countries with salt taxes. We then contacted the expert network associated with the review, including academics and organizations working to reduce population salt intake, to request information on any more recent examples of real-world salt taxation and/or any impact evaluations not captured in the 2015 review or reported in the peer-reviewed literature.

### Inclusion and exclusion criteria

Studies of any design were included if they provided information (i.e., empirical or modeled data) relevant to the implementation or evaluation of: *1*) taxes on salt that aimed to reduce salt intake; *2*) taxes on foods high in salt, including but not limited to processed foods and restaurant or fast foods; and *3*) taxes on unhealthy foods where the definition includes foods high in salt. Taxes on unhealthy foods where high salt or high sodium was not included as a criterion were excluded, because these have been evaluated elsewhere (18).

### Study selection

Two review authors (RD and MT, or RD and JAS) independently screened the titles and abstracts of the articles identified from the searches. The full texts of potentially eligible studies were obtained and assessed further by 2 review authors (RD and JAS). Disagreements were resolved through discussion and consultation with a third review author (JW).

### Data extraction and analysis

The following data were extracted from each study, by 2 review authors (RD and JAS): author, year of publication, country of study, intervention details (type of tax, level of implementation, alone or in combination with other interventions), method of evaluation (empirical or modeled data), outcome measures, and summary of results. Discrepancies in data extraction were resolved through discussion.

Where applicable, the quality of the included studies was assessed using the Critical Appraisal Skills Programme (CASP) checklist (available for free at https://casp-uk.net/casp-tools-checklists/). We assessed the quality of the studies that evaluated the effects of salt taxes, according to study type. Specifically, we used the CASP Economic Evaluation Checklist for modeling studies (27); the CASP Cohort Study Checklist for studies reporting real-world evaluation of impact (28); the CASP Randomised Controlled Trial Checklist for experimental studies (29); and the CASP Systematic Review Checklist for systematic reviews (30). Two authors (RD and JAS) independently conducted the assessments, and any disagreements were resolved through consultation with a third author (JW).

Data synthesis was based on all included studies. Due to the range of study designs, characteristics, and variation in quality, a meta-analysis could not be performed. Instead, a narrative synthesis of findings was deemed the most appropriate way to assess and report the evidence.

## Results

### Search results and quality assessment

**FIGURE 1:**
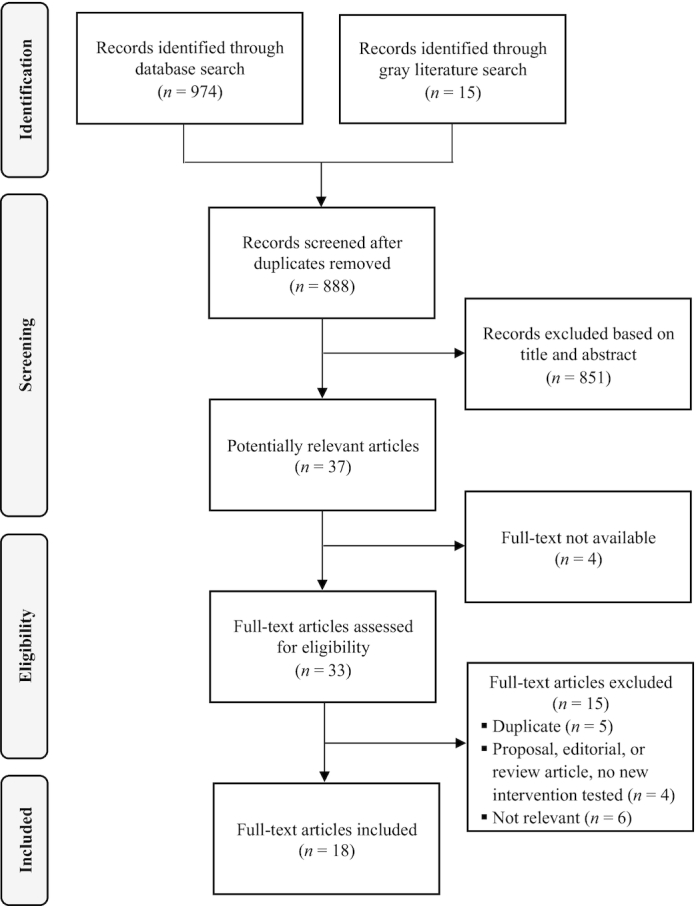
Flowchart of included studies.

The search identified 974 records from the peer-reviewed literature, and 15 additional records from the gray literature or through contact with experts. After removing duplicates, 888 abstracts were screened, of which 37 were considered potentially relevant (**Figure 1**). Of these, 4 full texts from the peer-reviewed literature were unavailable (2 were conference abstracts and 2 could not be found on any online database), and 15 were excluded after full-text screening for the following reasons: duplicate (*n* = 5); proposal, editorial, or review article with no new intervention tested (*n* = 4); and not relevant or the main focus was other forms of salt reduction interventions (*n* = 6). Ultimately, 18 studies met our inclusion criteria, of which 3 were gray literature studies. Of these, 15 studies evaluated the effects of salt taxes, either through modeling (*n* = 8) (31–38), real-world evaluation of impact (*n* = 4) (39–42), experimental study (*n* = 2) (43, 44), or review of cost-effectiveness and cost-utility (*n* = 1) (45). Two studies reported on consumer attitudes and stakeholder perceptions toward taxation of high-salt foods (42, 46). Lastly, 6 studies provided information relevant to implementation of salt taxes in 5 countries (15, 39–42, 47), including the 4 real-world evaluation studies.

**Supplemental Table 2** summarizes our quality assessment of the studies that examined the effects of salt taxes (*n* = 15). Overall, the modeling studies and the systematic review of cost-effectiveness studies met the quality criteria in most or all domains. Five modeling studies did not provide enough information to assess whether incremental or sensitivity analyses were conducted, or discounting was taken into account in the analysis. The real-world evaluation studies—mostly gray literature studies—also met the majority of quality criteria; however, there were also some important gaps. Three of the 4 studies did not report a measure of uncertainty (e.g., 95% CI or SE), making it impossible to judge the precision of results. In addition, the implications of these real-world studies for policy and practice, and their consistency with other available evidence, were scored “unclear,” though this could reflect the limited evidence base on salt taxation. Finally, the 2 simulation studies showed imprecise results (i.e., wide CIs), and, as simulation studies, the applicability of their results was unclear.

### Evidence of country implementation

**Table 1** lists the countries that have implemented fiscal measures to influence consumption of foods high in salt. In all 5 countries, these measures were part of a broader suite of tax measures designed to improve diet. For example, in Tonga taxes were also applied to fatty meats; Hungary also taxed foods high in fat and sugar; Fiji also taxed palm oil; and Saint Vincent and the Grenadines also placed value added tax on sugar and other sweetened beverages. Three of the 5 countries are small island states, and 4 are upper-middle-income countries (48).

**TABLE 1:** Countries with salt tax^1^

Reference	Country	Year initiated	Salt/sodium tax type and details
(39)	Hungary	2011	• Public Health Product tax: tax applied on a range of unhealthy foods including salty snacks and condiments that exceed recommended salt threshold levels
			salty snacks: if salt content >1 g/100 g, tax amount of HUF 250/kg (US${\$}$ 0.89/kg)
			condiments: if salt content >5 g/100 g, tax amount of HUF 250/kg (US${\$}$ 0.89/kg)
			mustard, ketchup, and nondehydrated, chopped or mashed salty vegetable flavorings:
			if salt content >15 g/100 g, tax amount of HUF 250/kg (US${\$}$ 0.89/kg)
(15, 40)	Fiji	2012	• Fiscal measures to promote healthy diet in 2012 budget include:
			import fiscal duty on MSG increased from 5% to 32% (applied to kilogram bags of MSG, not foods high in MSG)
(41)	Mexico	2013	• Eight percent tax on “nonessential” foods, including salty snacks, sweets, nut butters, cereal-based prepared products, that surpass a calorie density threshold (>275 cal/100 g)
			• Taxed salty snacks include potato chips, corn chips, flour chips, fried pork skin, ready-to-eat popcorn, microwave popcorn, crackers, peanuts, and seeds
(42)	Tonga	2015	• Excise tax of T${\$}$ 1/kg (US${\$}$ 0.45/kg) on imported instant noodles introduced in FY 2015–16 (replacing a customs duty), doubling to T${\$}$ 2/kg (US${\$}$ 0.90/kg) in FY 2017–18. A consumption tax of 15% also applied
(47)	Saint Vincent and the Grenadines	2016	• Value-added tax of 15% on salt, sugar, and other sweetened beverages

1 FY, fiscal year; HUF, Hungarian forint; MSG, monosodium glutamate; T${\$}$, Tongan Pa'anga.

### Effects of salt taxes

#### Findings from modeling studies

**Table 2** presents a summary of the 8 modeling studies that evaluated the impact of salt taxes. All studies were from high-income countries based on the World Bank income classification (48)—2 each from the United States (32, 37), New Zealand (34, 35), and the United Kingdom (33, 36), and 1 each from Australia (31) and Chile (38). A ninth review study (45) considered the relative cost-effectiveness of salt reduction initiatives across a range of modeling studies, including salt tax studies.

**TABLE 2:** Effects of salt taxes: modeling studies^1^

Reference	Author, year, and country	Intervention details	Methods of evaluation	Outcome measures	Summary of results
(31)	Cobiac et al., 2017Australia	Salt tax: A${\$}$ 0.30/g of sodium in excess of Australian maximum recommended levels, excluding fresh fruits, vegetables, meats, and dairy products.Other interventions tested: Saturated fat tax of A${\$}$ 1.37/100 g Sugar-sweetened beverage tax of A${\$}$ 0.47/L Fruit and vegetable subsidy of A${\$}$ 0.14/100 g Sugar tax of A${\$}$ 0.94/100 mL of ice cream and A${\$}$ 0.85/100 g of sugar	Modeling study using price survey data from Australian supermarkets; price elasticity data and a proportional multistate lifetable model	Changes to dietary intake and dietary cost DALYS averted Cost-savings from reduced disease burden Cost-effectiveness of each intervention modeled	Excess salt tax would reduce salt consumption by 67 mg/d and total energy intake by 161 kJ/d. Marginally negative effect on fruit and vegetable intake. Negligible impact on food costs Estimated 130,000 DALYS averted (total population 22 million) saving A${\$}$ 2 billion in health costs Sugar tax resulted in the largest health gain (270,000 DALYs averted) and cost savings (A${\$}$ 4 billion), followed by salt Combination of taxes and subsidy could avert 470,000 DALYs with a net cost saving of A${\$}$ 3.4 billion to the health sector Excess salt tax led to a “dominant” cost-effectiveness (i.e., increase in net health gain and cost-savings)
(32)	Harding and Lovenheim, 2017 United States	Salt tax: 20% tax applied to products based on their salt content, across 14 product groups.Other interventions tested: 20% product-specific tax: applied on products considered to be unhealthy 20% nutrient-specific tax: applied based on a product's fat and sugar content 20% sugar-sweetened beverage tax: applied on products that have large sugar content	Modeling study based on transaction-level data from a large sample of US consumers and product-specific nutrient information	Changes in consumer expenditures Changes in nutrient content of purchased products Welfare cost of tax	The 20% salt-specific tax was estimated to reduce the total salt purchased by households by 10.0%/mo, which is equivalent to 18,792 mg of salt. Across the 14 product groups, the salt tax was estimated to reduce the salt purchased in 12 groups, ranging from 35.5 mg (baking goods) to 4591.1 mg (canned foods and sauces). The utility cost of this tax was low, at 1.2% (US${\$}$ 10.95) The tax was estimated to also reduce the calories, total fat, and sugar purchased by households monthly by 10.63%, 10.51%, and 1.53%, respectively
(38)	Caro et al., 2017 Chile	Salt tax: stand-alone salt tax was not considered. Other interventions tested: 18% price tax on foods and beverages high in fat, salt, and added sugar 40% sugar-sweetened beverage tax 1 Chilean peso per gram of sugar tax on products with added sugar	Modeling study looking at changes in nutrient availability, based on income and expenditure data survey using a utility-based structural model	Changes in nutrient availability based on nutrient content of household food purchases, stratified by household income	Scenarios 1 and 3 led to significant reductions in sodium from all food groups, by 22.52 mg and 11.49 mg, respectively Of the 3 scenarios, the 18% tax produced the largest reductions in calories, carbohydrates, sugar, sodium, total fats, and saturated fats from the “junk” food group (i.e., sweets and desserts, salty snacks and chips, ready-to-drink SSBs, and SSBs from concentrates) The changes in nutrient availability for both low-income and mid-to-high-income households were comparable, and consistent with the overall results
(34)	Nghiem et al., 2015 New Zealand	Salt tax: excise tax on salt increased progressively by ≤20% annually to achieve a salt intake level of 2300 mg/d.Other interventions tested: Dietary counseling by dietitians Endorsement label program Mandatory reduction of salt in breads, processed meats, and sauces Mandatory reduction of salt in processed foods by 25% UK Package: media campaigns, voluntary food reformulation, and food labeling Mass media campaign component of the UK Package Reduction in the amount of food-grade salt released to NZ market	Modeling and cost-utility analysis using Markov model, based on a cohort of 2.3 million adults aged >35 y	Health gain measured by QALYs Cost-savings	Salt tax was associated with 195,000 QALYs gained, and produced discounted net savings of NZ${\$}$ 1.0 billion over the remainder of the cohort's life, due to CVD treatment costs averted The tax was estimated to produce NZ${\$}$ 452 million in revenue per year
(35)	Ni Mhurchu et al., 2015 New Zealand	Salt tax: 20% tax on major dietarysources of sodium Combination of 20% tax on major sources of sodium and saturated fat, and 20% subsidy on fruit and vegetables. Other interventions tested: 20% subsidy on fruit and vegetables 20% tax on major dietary sources of saturated fat 20% tax on major food contributors to carbon emissions	Modeling study looking at dietary intake and resulting effects on diet-related diseases using household expenditure data and demand elasticities	Changes in food and nutrient purchases Deaths prevented or postponed Equity effects	The sodium tax was estimated to reduce the total energy and sodium purchases by 7.17% and 10.77%, respectively, but increase saturated fat purchases by 2.19%. The combination of taxes (sodium and saturated fat) and subsidy (fruit and vegetables) reduced household purchases of total energy, sodium, and saturated fat by 6.15%, 10.79%, and 3.64%, respectively The sodium tax and the combination scenario were associated with 1977 and 2352 lives saved per year, respectively, mostly from deaths averted due to CVDs Effects were equal or larger in low-income and Maori households
(37)	Smith-Spangler et al., 2010 United States	Salt tax: excise tax on sodium used for food production at the industrial level, which would increase the price of salty foods by 40%.Other interventions tested: collaboration with food industry through voluntary reduction in sodium content of processed foods	Modeling study using Markov model to assess cost-effectiveness of the interventions	Health consequences measured by strokes and MIs averted, QALYs gained, and life-years gained Cost savings	The sodium tax was estimated to avert 327,892 strokes and 306,137 MIs over the lifetime of adults aged 40–85 y, leading to ∼840,113 LGYs and 1.3 billion QALYs gained, and saving US${\$}$ 22.4 billion in direct medical costs
(36)	Nnoaham et al., 2009 United Kingdom	Salt tax: stand-alone salt tax was not considered.Other interventions tested: 17.5% VAT on major dietary sources of saturated fat 17.5% VAT on foods defined as less healthy by WXYfm nutrient profiling (includes foods high in salt) Scenario 2 combined with 17.5% subsidy on fruit and vegetables Scenario 2 combined with 32.5% subsidy on fruit and vegetables	Modeling study based on national data on food expenditure and consumption	Changes in food and nutrient purchases Changes in annual deaths from CHD, stroke, cancer, and CVD Economic impact on households (by income quintile)	Scenarios 2, 3, and 4 reduced average salt consumption by 1.86%, 1.10%, and 0.45%, respectively. Scenario 1 led to a small increase in salt intake, of 0.24% Scenarios 1 and 2 marginally increased total deaths annually, due to falling intake of fruit and vegetables that outweighs impact of reduced consumption of unhealthy foods Scenarios 3 and 4 prevented 1563–2870 and 3689–6435 deaths annually, a relatively small number in the UK context All 4 scenarios are economically regressive, with the poorest households absorbing proportionally higher costs than richer households. By contrast, the health gain is even across income quintiles
(33)	Mytton et al., 2007 United Kingdom	Salt tax: stand-alone salt tax was not considered.Other interventions tested:To extend 17.5% VAT to Main sources of saturated fat Foods classified as unhealthy using SSCg3d model (includes high salt) A targeted “best outcome” package of unhealthy foods identified by the authors	Modeling study based on consumption, expenditure, and elasticity data from National Food Survey	Changes to household food expenditure Changes to consumption, including salt intake Changes to mortality for IHD, stroke, and overall CVD deaths	The 3 scenarios predicted a decrease in fruit and vegetable intake by 1.2–3.9%, and increase in household food expenditure by 3.2–4.6% Scenario 1 increased salt intake by 5.2%, whereas scenarios 2 and 3 reduced salt consumption by 5.8% and 6.6%, respectively Scenario 1 resulted in more IHD, stroke, and overall CVD deaths. Scenario 2 predicted 2100–2500 fewer deaths annually, primarily due to reduction in salt intake. Scenario 3 produced a larger reduction in CVD deaths (2600–3200), due to reduced salt intake
(45)	Schorling et al., 2017 OECD countries	Salt tax: Nghiem et al. (34) and Smith-Spangler et al. (37) modeling studies, included above.Other interventions tested: voluntary and mandatory salt substitution and reduction, dietary advice, labeling, and awareness campaign	Review of cost-effectiveness and cost-utility analyses of salt reduction studies	Changes in salt intake and SBP Incremental benefit as QALYs, DALYs, or life-years gained	Fourteen studies covering 62 scenarios were found, of which 59 were cost-saving The ICER for salt taxation was Intl.${\$}$ 19,000 per QALY or Intl.${\$}$ 29,702 per LYG (Smith-Spangler) or Intl.${\$}$ 3660 per QALY (Nghiem) By comparison, the ICER for other interventions was Intl.${\$}$ 7–109,324 per QALY, with salt taxation, voluntary and mandatory salt reduction in processed foods, and labeling scoring especially low ICER. Targeted dietary advice was cost-ineffective

1 A${\$}$, Australian dollar; CHD, coronary heart disease; CVD, cardiovascular disease; DALY, disability-adjusted life year; ICER, incremental cost-effectiveness ratio; IHD, ischemic heart disease; Intl.${\$}$, International dollar; LYG, life-year gained; MI, myocardial infarction; NZ${\$}$, New Zealand dollar; QALY, quality-adjusted life year; SBP, systolic blood pressure; SSB, sugar-sweetened beverage; SSCg3d, simple scoring system, group C nutrients, per 100g; VAT, value added tax; WXYfm, United Kindgom Food Standards Agency/Of com model.

These studies present 3 main approaches to modeling salt taxation:

- Taxing salt itself: 2 studies did this, either through an excise tax on salt (34) or industrial sodium (37), on the assumption that food producers will pass on these costs, and high-salt foods will become more expensive. A third study (31) modeled a tax per gram of excess salt, which means that the level of taxation is precisely calibrated to the amount of salt used.
- Taxing foods high in salt: 2 studies (32, 35) applied a 20% tax on foods that exceed a salt threshold. All foods were taxed at the same rate, regardless of their level of excess salt.
- Taxing unhealthy foods: 3 studies applied sales tax of either 17.5% (33, 36) or 18% (38) on foods defined as unhealthy using a nutrient profile that included, but was not limited to, high salt.

The majority of studies (6/8) modeled the impact on salt consumption, by estimating either household purchases of food high in salt, or individual salt intake. Only 1 modeling study included the assumption that the food industry would respond to taxation with reformulation (31); 3 others recognized such a response was possible, but did not model it (32, 35, 38). Across the studies, results were consistently positive, but of varying magnitude and difficult to compare given the range of measures used. A 20% tax on products high in salt was predicted to reduce monthly household salt purchases by 10% in the United States (32) and 11% in New Zealand (35). In contrast, a tax of A${\$}$ 0.3/g of excess salt was predicted to reduce salt intake by 67 mg/d (31) in Australia (percentage decline not provided). Studies modeling taxes on unhealthy foods (as defined by a nutrient profile encompassing salt, sugar, fat) also had varying results. The 17.5% tax rate applied by the UK studies predicted reductions of 1% (36) and 6% (33) in salt consumption, whereas the 18% tax modeled in Chile was estimated to reduce salt intake by 22.5 mg/adult/d (percentage decline not provided).

Six of the 8 studies modeled the health gain associated with estimated reduced salt consumption, expressed either as quality-adjusted life years (QALYS), DALYS, or deaths averted. The magnitude of the health gain was proportional to the modeled drop in salt intake (in those studies that modeled both), and was found to produce health gains in all studies except 1 (36). A tax on excess salt averted 130,000 DALYS in Australia (31), which has a population of 22 million, whereas in New Zealand (34) a more aggressive taxation regime resulted in 195,000 QALYS gained in a population of <5 million. Also in New Zealand (35), a 20% tax on foods high in salt was estimated to avert almost 2000 deaths annually; however, a UK study (33) found that a similar level of taxation on foods classified as unhealthy (including high-salt foods) resulted in a similar total number of deaths averted (2100–2500), despite the United Kingdom's much larger population.

The UK study by Nnoaham et al. (36) produced contrasting findings. The study suggested that taxing foods defined as less healthy (using a nutrient profile model) might marginally increase total deaths, based on the assumption that higher costs of some (taxed) food would crowd out spending on fruit and vegetables. Other studies also highlighted the potential impact of cross-price elasticities and product substitution—both negative and positive. For example, a US study (32) found that nutrient taxes targeting sugar and fat have a similar impact on salt consumption as a dedicated salt tax, likely because many foods, especially junk foods, that are high in sugar are also high in salt. A New Zealand experimental study (44) had similar outcomes (see Table 2), finding that salt taxation led to a 4.3% increase in the proportion of fruit and vegetables purchased, but also a 3.2% increase in total sugars as a percentage of total energy purchases.

Three studies considered the cost savings for the health systems based on death and disability averted, and 1 of these also calculated revenue raised from the introduction of a salt tax. Again, the magnitude varied—due to both the size of the country and the type of tax applied—but all findings were positive. Two studies looked at cost-effectiveness (the cost of implementing the intervention compared with the health gain), both finding net positive effects (34, 37). The review (45) compared salt tax with other salt reduction interventions, finding it on a par with voluntary and mandatory salt reduction in packaged foods in having a “specially low cost-effectiveness ratio,” that is, highly cost-effective.

Just 3 studies—from New Zealand (35), the United Kingdom (36), and Chile (38)—considered equity. The New Zealand and UK studies found health gains to be evenly spread or progressive, although the latter (36) found food taxes to be economically regressive. In Chile (38), changes in nutrient intake were comparable between low- and middle-to-high-income households. Finally, 3 studies compared the introduction of taxes (on their own) with policies of tax plus subsidies for fruit and vegetable. All found that the latter delivered the largest health gain (31, 35, 36).

#### Findings from real-world evaluation of impact

Four real-world studies looked at the impact of salt taxes, 3 of which were gray literature studies (**Table 3**). Two studies (from Hungary and Tonga) evaluated the impact of taxation measures on personal consumption, both reporting modestly positive results. In Hungary, 11–16% of those consuming salty snacks and condiments reported changing their behavior due to the tax, but of these, only 5% switched to healthier alternatives. The majority switched to cheaper brands, and overall levels of salty food consumption remained high (39). Tonga recorded steep declines in the import of instant noodles in the year after excise tax introduction, and the following year 30% of those surveyed reported reducing their consumption of instant noodles. However, the World Bank modeling and qualitative surveys suggest that the level of reduction was small. Further, locally manufactured instant noodles, which are not subject to excise tax, became a key substitute for imported instant noodles (42). In both countries, taxes had a greater impact on reducing consumption of other types of unhealthy foods—notably foods high in sugar—possibly due to the low base price of salty foods. Low base price was likely to have been a factor in Fiji as well, where the monosodium glutamate (MSG) tax had limited impact, with imports of the product rising in the years after tax introduction.

**TABLE 3:** Real-world evaluation of impact of salt taxes^1^

Reference	Author and year	Country	Evaluation period	Method of evaluation	Summary of results
(39)	World Health Organization Regional Office for Europe, 2015	Hungary	2011 to 2014	Data on consumption of products with public health tax were obtained through a questionnaire and a 3-d nutrition diary Data on tax revenue were obtained from the National Tax and Customs Administration	Proportion of adults consuming taxed salty snacks rose, from 69% to 71%; and in 2014 78% still bought salty condiments (no comparative data) Of those consuming salty snacks or condiments, 16% and 11%, respectively, changed behavior by either reducing consumption or switching to cheaper brands Under 5% substituted other foods, but the majority of these chose healthier foods Salty snacks and condiments contributed 30% of tax revenue gained
(40)	Pacific Research Centre for the Prevention of Obesity and NCDs, 2017	Fiji	2011 to 2013	Data on import volume (used as proxy for consumer consumption) were obtained from the Fiji Bureau of Statistics	Volume of MSG imports increased, rising from <50,000 kg in 2011 to >200,000 kg in 2013
(41)	Taillie et al., 2017	Mexico	2012 to 2015	Data on household food purchases were obtained from the Nielsen Company's Mexico Consumer Panel Services	Taxed food purchases declined by 6.0% within 2 y of tax introduction, accelerating in year 2 Low socioeconomic households were more likely to decrease their purchases of taxed foods, suggesting pro-equity effects, as were households that had shown greater preference for taxed foods pretax No disaggregated data on consumption of salty snacks or reduction of salt intake
(42)	The World Bank, 2019	Tonga	2014–15 to 2017–18	Data on import volumes and revenues were obtained from government administrative data Data on consumption and behaviors were obtained through household surveys (questionnaire) Data on prices of products were obtained through retail surveys	The average retail price of instant noodles increased from T${\$}$ 0.88 per pack in FY2014–15 to T${\$}$ 1.14 per pack in FY2017–18 Sharp decrease in imports of imported noodles from 2,083,000 kg in 2014–15 to 439,000 kg in 2016–17. However, the volume rebounded to 806,000 kg in FY 2017–18, even though the excise tax doubled 70% of those surveyed did not change consumption of instant noodles as a result of the price change, likely due to the low base price As a result of the excise tax on fatty meats, 6–7% stopped eating turkey tails and mutton flaps products altogether, whereas 22% switched to a cheaper alternative to turkey tails and 40% looked for cheaper alternatives to mutton flaps, respectively), the most frequent of which was salted beef

1 FY, fiscal year; MSG, monosodium glutamate; T${\$}$, Tongan Pa'anga.

Mexico has reported significant gains from its nonessential food taxes, with sales of taxed, unhealthy products falling by 6% in 2 y. However, there is not yet any specific, published information on intake of high-salt products. Experience from Tonga and Hungary suggests these disaggregated data are needed because declines in sugar and fat intake are not necessarily replicated for salt. Indeed, as in the modeling studies, there is some evidence that product substitution could have unintended negative effects on salt consumption: Tonga found that taxes on fatty meats (turkey tails, mutton flaps) led to substitution with salted beef and corned beef, which were exempt from taxation.

#### Findings from experimental studies

Two experimental studies looked at the impact of price increases and subsidies on food purchases using a simulated online supermarket (**Table 4**). The results align with the findings of modeling studies, that nutrient-based taxation is likely to have a larger effect than product-based taxation, but also that substitution effects can have unintended, perverse consequences.

**TABLE 4:** Effects of salt taxes: experimental studies^1^

Reference	Author, year, and country	Intervention details	Methods of evaluation	Outcome measures	Summary of results
(44)	Waterlander et al., 2019 New Zealand	Salt tax: NZ${\$}$ 0.02/100 mg and NZ${\$}$ 0.04/100 mg Na.Other interventions tested: Fruit and vegetable subsidy, 20% Sweetened beverage tax, 20% and 40% Saturated fat tax, NZ${\$}$ 2.0/100 g and NZ${\$}$ 4.0/100 g Sugar tax, NZ${\$}$ 0.4/100 g or NZ${\$}$ 0.8/100 g	Experimental study based on ≤5 weekly shops in a virtual supermarket	Healthiness of the total shopping basket Percentage change in purchases of taxed goods Substitution effects	For the weekly household shop, salt taxes led to 10.7 g mean decrease in salt purchased (averaged across both modeled taxes) Saturated fat tax, sugar tax, and salt tax resulted in small but significant increases in the proportion of healthy food in the weekly shop (1.8%, 1.1%, and 1.3%, respectively). Other interventions had no impact on this outcome Salt tax led to a 4.3% increase in proportion of fruit and vegetables in the weekly shop, but also a 3.2% increase in sugar as a percentage of total energy purchases. Saturated fat tax had a similar effect
(43)	Epstein et al., 2015 United States	Salt tax: stand-alone salt tax was not considered.Other interventions tested: a range of healthy and unhealthy foods (including salty foods) were taxed at a rate of 12.5% and 25%, or subsidized at rates of 12.5% or 25%	Experimental study using simulated online supermarket	Changes to purchases of foods taxed or subsidized Changes to overall weekly food basket Changes to total calories and nutrients purchased	Application of subsidies improved overall nutrient intake but led to a negligible change in salt consumption Taxes had no overall impact on nutrition profile, and also led to negligible change in salt intake

1 NZ${\$}$, New Zealand dollar.

The New Zealand study (44) used a nutrient-based approach to taxing salt, that is, calibrating the price increase to the amount of salt in food. This led to a 10.7-g mean decrease in salt purchased in the weekly shop, as well as an increase in fruit and vegetable purchases, but also a small rise in total sugar purchases. In contrast, the US study (43) modeled the impact of taxing a range of unhealthy foods (including salty foods) at different rates, and subsidizing healthy foods. It found taxes had no overall positive impact on nutrition profile of the weekly shop, and marginally increased salt intake from salty snacks not covered by taxes.

### Perceptions of taxation of high-salt foods

Two studies reported on consumer attitudes toward salt taxation. In Ireland (46), salt tax was the least popular of proposed salt reduction initiatives, though 42% of those surveyed were in favor. Support for salt taxation was highest amongst those who saw food manufacturers as responsible for reducing salt consumption, suggesting knowledge of the food production process could be key to winning public support. Similarly, in Tonga, focus group discussions revealed food taxes to be unpopular with consumers, due to the cost for consumers, but also to limited knowledge that the purpose of the tax was to promote healthy eating (42).

## Discussion

Our literature search found some evidence on the potential effectiveness and cost-effectiveness of salt taxation, but the bulk of evidence comes from modeling studies, and results vary considerably depending on the parameters of the modeling. Further, the majority of empirical or real-world evidence comes from the gray literature; our quality assessment found the overall quality of these studies to be moderate, but raised questions on the precision of their results (Supplemental Table 2).

Taxes calibrated to the level of salt content (a type of “nutrient-specific” tax) deliver the strongest results in terms of reduced salt intake, largely due to their broad application. Such taxes are applied to all foods containing salt (or in some cases, excess salt) reducing options for substitution, and are adjusted to the level of salt content, making those products highest in salt most expensive. Nutrient-specific taxes can also be designed to consider the overall nutrient profile. Modeling suggests this approach delivers good results in terms of overall diet improvement—typically measured in terms of calorie intake. The reported impact on salt consumption is variable; this could be an underestimate given that most modeling studies did not account for reformulation, which evidence from sugar tax implementation suggests is a likely industry response (49). Finally, both real-world and modeling studies reported that taxation leads to both positive and negative product substitution.

Our findings are in line with a growing body of literature on the effectiveness of fiscal measures—taxation and subsidy—in encouraging healthy diet (18, 19). However, we found limited uptake of salt tax in practice: all “real-world” examples of salt taxation we identified involved taxing specific products, such as salty snacks, rather than taxing all foods with a salt content above a certain threshold (i.e., a nutrient-specific tax). Overall, taxation of salt or salty food is much less common than taxation of products high in sugar, which has been introduced in ≥35 countries (50) along with several US cities (44).

Although no studies we found explored reasons for the lack of uptake for salt tax, we speculate that a possible reason is that salt tax can be more difficult to apply in practice. Many sugar taxes have a narrow scope, targeting single products such as sugar-sweetened beverages (SSBs), making them relatively straightforward to implement. In most countries there is no natural equivalent for salt—no single “salty snack”—and salt tends to be pervasive throughout the food supply. As such, salt tax is typically part of a broader suite of unhealthy food measures, rather than a stand-alone initiative, and these approaches are more challenging both technically and politically.

Equally, the lack of real-world evidence can present something of a “catch-22” (36) making it hard to build a case for salt tax introduction, in turn resulting in limited evidence. By contrast, the evidence base for sugar taxation, especially in relation to SSBs, is large and growing, helping to build momentum for wider application (51).

Policy-makers might also fear public backlash. Evidence we found on the unpopularity of salt tax is in line with other studies (52) and those showing low “willingness to pay” for salt reduction programs (53). Indeed, a recent, failed attempt to introduce salt tax in the Philippines shows how politically challenging the issue can be (54, 55). Conversely, public support is likely to be greater for measures already implemented, compared with those proposed (56)—again pointing to the catch-22 of lack of real-world evidence hampering implementation.

Those countries we found that *are* experimenting with salt tax are predominantly upper-middle-income countries (4/5) and/or small island states (3/5); in all, consumption of processed foods is growing and is a key source of salt intake (57, 58). Salt tax could be of less interest to low-income countries given that the major source of sodium is added salt during cooking, and the low base price of salt means even a large tax leads to only a small price increase. Nghiem et al. (34) found that an excise tax on salt in New Zealand would need to be applied at a rate of 20%/y, for 10 y, in order to reduce salt intake to desired levels. Similarly, in Fiji, the 32% tax on kilo bags of MSG had no impact, in part due to the low base price. Taxing standard salt (sodium chloride) while subsidizing reduced-sodium salts (made by substituting some of the sodium for potassium) might be an option in these contexts (59).

Our study points to a number of gaps in the evidence base that could warrant further attention. First, there are few real-world impact studies. Second, when impact studies are undertaken, they do not always track impact on all aspects of diet (e.g., they might measure changes in calorie intake but not salt consumption). Third, most real-world studies we found did not use a comparable control or counterfactual to account for changes that would have happened in the absence of a tax. Fourth, there is limited qualitative evidence on, for example, public attitudes toward salt taxation, and we found no studies that examined the political context and drivers that might influence policy. Finally, further exploration of the equity effects of salt tax is required, given conflicting evidence from the few studies done to date.

The key strength of our study was that we conducted a comprehensive search of the published literature alongside an extensive review of real-world evidence, including gray literature, allowing us to assess both the effectiveness and feasibility of salt taxation. Our key limitation is that we were unable to perform a meta-analysis of the data presented, due to variations in methodology, quality, taxation model, and the choice of impact indicators.

## Conclusion

This study found some positive, theoretical evidence on the potential for fiscal policies to reduce salt consumption and improve diet, but limited “real-world” evidence on the impact of salt tax in practice due to limited use to date. Where such taxes have been introduced, they are more likely to be applied to particular products (such as instant noodles) rather than to all foods over a certain salt threshold. Experience with sugar taxation also suggests that product-specific approaches are more feasible both politically and technically. However, modeling studies suggest that comprehensive approaches, which target a broader range of unhealthy foods, are likely to yield greater benefits, and minimize opportunity for substitution. There is strong evidence for the need to reduce salt intake so further consideration on the use of fiscal measures to reduce salt intake is warranted.

## Supplementary Material

nmaa067_Supplemental_Files

## Notes

The authors reported no funding received for this study.

Author disclosures: JW is the Director of the WHO Collaborating Centre on Population Salt Reduction, is supported by a National Heart Foundation Career Development Fellowship (1082924), and through an NHMRC Centre of Research Excellence on food policy interventions to reduce salt (1117300). KT is supported by an NHMRC Early Career Fellowship (1161597) and a Postdoctoral Fellowship (102140) from the National Heart Foundation of Australia. JAS is supported by an NHMRC Postgraduate Scholarship (1168948). NRCC was a paid consultant to the Novartis Foundation (2016–2017) to support its program to improve hypertension control in low- to middle-income countries, which included travel support for site visits and a contract to develop a survey. NRCC has provided paid consultative advice on accurate blood pressure assessment to Midway Corporation (2017) and is an unpaid member of World Action on Salt and Health (WASH). FJH is a member of Consensus Action on Salt and Health (CASH) and WASH; both are nonprofit charitable organizations and FJH does not receive any financial support from them. All other authors report no conflicts of interest.

Supplemental Tables 1 and 2 are available from the “Supplementary data” link in the online posting of the article and from the same link in the online table of contents at https://academic.oup.com/advances.

Abbreviations used: CASP, Critical Appraisal Skills Programme; DALY, disability-adjusted life years; MSG, monosodium glutamate; NCD, noncommunicable disease; QALY, quality-adjusted life year; SSB, sugar-sweetened beverage.
