# Supplementary material for: Effectiveness and Feasibility of Taxing Salt and Foods High in Sodium: A Systematic Review of the Evidence
Source: Adv Nutr. 2020 Jun 20;11(6):1616–30. doi: 10.1093/advances/nmaa067 (PMC7666895; doi:10.1093/advances/nmaa067)
Supplement: nmaa067_Supplemental_Files [file nmaa067_supplemental_files.zip › Supplementary data 1.docx]

**Supplemental Table 1:** Search strategy (MEDLINE)

| **No** | **Searches** |
| --- | --- |
| 1 | sodium, dietary/ or sodium chloride, dietary/ |
| 2 | Sodium Chloride/ |
| 3 | Diet, Sodium-Restricted/ |
| 4 | ((salt or sodium) adj10 (reduc* or target* or cutback* or decreas* or limit* or consumption)).tw. |
| 5 | ((diet* or nutrition* or food or intake) adj10 (salt or sodium)).tw. |
| 6 | 1 or 2 or 3 or 4 or 5 |
| 7 | taxes/ or tax exemption/ |
| 8 | (tax* not ("taxi" or "taxonomy" or taxa* or taxo*)).tw. |
| 9 | financing, organized/ or financing, government/ |
| 10 | "Cost Sharing"/ |
| 11 | (pric* or fiscal or penalty or penalties or levy or levied or excis*).tw. |
| 12 | (taxation or taxes or subsid*).tw. |
| 13 | (financial adj3 (incentive* or disincentive*)).tw. |
| 14 | 7 or 8 or 9 or 10 or 11 or 12 or 13 |
| 15 | 6 and 14 |
| 16 | exp animals/ not humans.sh. |
| 17 | 15 not 16 |
| 18 | limit 17 to yr="2000-Current" |
