# Supplementary material for: Effectiveness and Feasibility of Taxing Salt and Foods High in Sodium: A Systematic Review of the Evidence
Source: Adv Nutr. 2020 Jun 20;11(6):1616–30. doi: 10.1093/advances/nmaa067 (PMC7666895; doi:10.1093/advances/nmaa067)
Supplement: nmaa067_Supplemental_Files [file nmaa067_supplemental_files.zip › Supplementary data 2.docx]

**Supplemental Table 2:** Quality assessment of included studies

A. Quality assessment of the modelling studies^[[1]](#footnote-1)^

| **Ref** | **Study** | **Is the economic evaluation valid?** | | | | **How were consequences and costs assessed and compared?** | | | | | **Will the results help in purchasing for local people?** | | |
| --- | --- | --- | --- | --- | --- | --- | --- | --- | --- | --- | --- | --- | --- |
|  |  | *Was a well-defined question posed?* | *Was a comprehensive description of the competing alternatives given?* | *Does the paper provide evidence that the programme would be effective?* | *Were the effects of the intervention identified, measured and valued appropriately?* | *Were all resources and health outcome costs for each alternative identified, measured, and valued credibly?* | *Were costs and consequences adjusted for different times at which they occurred (discounting)?* | *What were the results of the evaluation?* | *Was an incremental analysis of the consequences and costs of alternatives performed?* | *Was an adequate sensitivity analysis performed?* | *Is the programme likely to be equally effective in your context or setting?* | *Are the costs translatable to your setting?* | *Is it worth doing in your setting?* |
| (32) | Cobiac 2017 | Y | Y | Y | Y | Y | Y | Y | Y | Y | Y | Y | Y |
| (33) | Harding 2017 | Y | Y | U | Y | Y | U | Y | U | U | Y | Y | Y |
| (39) | Caro 2017 | Y | Y | Y | Y | Y | U | Y | U | Y | Y | Y | Y |
| (35) | Nghiem 2015 | Y | Y | U | Y | Y | Y | Y | Y | Y | Y | Y | Y |
| (36) | Ni Mhurchu 2015 | Y | Y | Y | Y | Y | U | Y | U | Y | Y | Y | Y |
| (38) | Smith-Spangler 2015 | Y | Y | U | Y | Y | Y | Y | Y | Y | Y | Y | Y |
| (37) | Nnoaham 2009 | Y | Y | U | Y | Y | U | Y | U | U | Y | Y | Y |
| (34) | Mytton 2006 | Y | Y | U | Y | Y | U | Y | U | Y | Y | Y | Y |

B. Quality assessment of the real-world studies

| **Ref** | **Study** | **Are the results of the study valid ?** | | | | | | | | **What are the results?** | | | **Will the results help locally?** | | |
| --- | --- | --- | --- | --- | --- | --- | --- | --- | --- | --- | --- | --- | --- | --- | --- |
|  |  | *Did the study address a clearly focused issue?* | *Were the participants recruited in an acceptable way?* | *Was the exposure accurately measured to minimise bias?* | *Was the outcome accurately measured to minimise bias?* | *Have the authors identified all important confounding factors?* | *Have the authors taken account of the confounding factors in the design or analysis?* | *Was the follow-up of subjects complete enough?* | *Was the follow-up of subjects long enough?* | *What are the results of this study?* | *How precise are the results?* | *Do you believe the results?* | *Can the results be applied to the local population?* | *Do the results of this study fit with other available evidence?* | *What are the implications of this study for practice?* |
| (40) | WHO-EURO 2015 | Y | Y | Y | U | U | Y | NA | Y | Y | U | Y | Y | U | U |
| (41) | C-POND 2016 | Y | Y | Y | U | N | N | NA | Y | Y | U | Y | U | U | U |
| (42) | Taillie 2017 | Y | Y | Y | Y | U | Y | Y | Y | Y | Y | Y | Y | U | U |
| (43) | World Bank 2019 | Y | Y | N | U | U | Y | Y | Y | Y | U | Y | Y | U | U |

C. Quality assessment of the experimental studies

| **Ref** | **Study** | **Are the results of the study valid?** | | | | | | **What are the results?** | | **Will the results help locally?** | | |
| --- | --- | --- | --- | --- | --- | --- | --- | --- | --- | --- | --- | --- |
|  |  | *Did the trial address a clearly focused issue?* | *Was the assignment of participants to treatments randomized?* | *Were all the participants who entered the trial properly accounted for?* | *Were participants and study personnel blind to treatment?* | *Were the groups similar at the start of the trial?* | *Aside from the experimental intervention, Were the groups treated equally?* | *How large was the treatment effect?* | *How precise was the estimate of the treatment effect?* | *Can the results be applied in your context?* | *Were all clinically important outcomes considered?* | *Are the benefits worth the harms and costs?* |
| (45) | Waterlander 2019 | Y | Y | Y | Y | NA | Y | N | N | U | U | U |
| (44) | Epstein 2015 | Y | Y | Y | U | NA | Y | N | N | U | U | U |

D. Quality assessment of the systematic review

| **Ref** | **Study** | **Are the results of the study valid?** | | | | | **What are the results?** | | **Will the results help locally?** | | |
| --- | --- | --- | --- | --- | --- | --- | --- | --- | --- | --- | --- |
|  |  | *Did the review address a clearly focused question?* | *Did the authors look for the right type of papers?* | *Do you think all the important, relevant studies were included?* | *Did the review authors do enough to assess quality of the included studies?* | *If the results of the review have been combined, was it reasonable to do so?* | *What are the results?* | *How precise are the results?* | *Can the results be applied to the local population?* | *Were all important outcomes considered?* | *Are the benefits worth the harms and costs?* |
| (46) | Schorling 2017 | Y | Y | Y | Y | NA | Y | NA | Y | U | Y |

1. N, no; NA, not applicable; Ref, reference; U, unclear; Y, yes. [↑](#footnote-ref-1)
